# Needs assessment to shape rural health research capacity building strategies: a mixed methods study

**DOI:** 10.3389/fmed.2026.1932638

**Published:** 2026-10-28

**Authors:** Belinda O’Sullivan, Anton Isaacs, Logan Westley, Cathy Haigh, Andrea Nicholson

**Affiliations:** 1 Monash University, School of Rural Health, Bendigo, VIC, Australia; 2 Monash University, School of Rural Health, Warragul, VIC, Australia; 3 Monash University, School of Rural Health, Traralgon, VIC, Australia

**Keywords:** rural health, capacity building, research, executive, clinician

## Abstract

**Introduction:**

Rural areas require tailored rural health research capacity building aligned to the local needs and interests. To set the directions for a rural health research capacity building strategy, we aimed to conduct a needs assessment of rural health research interests, barriers and enablers across rural health stakeholders.

**Methods:**

We conducted mixed methods research that included an anonymous online survey of Australian rural health stakeholders from hospitals, primary care and rural health agencies and qualitative interviews with those who consented from two rural regions in Victoria, Australia.

**Results:**

Forty individuals completed the survey and twenty-six individuals participated in interviews. The aggregate data suggested that there is strong interest in rural health research although many survey respondents preferred to be aware about research that is underway and which they could get involved in, rather than to lead it. Interview findings suggested that rural health research is considered useful for driving rural health investments, promoting quality healthcare and recruiting and retaining rural health workforces. Barriers to rural health research included higher workloads in smaller clinical teams, limited funding, under-developed research structures, organisation, time and tools. Enablers included having a research strategy aligned with the community's needs, research governance/planning processes for research teams, research tools and academic networks. The balance of interests, barriers and enablers for rural health research capacity building varied between rural health executives and clinicians but was consistent across hospitals and other rural health services.

**Conclusions:**

Rural health research is valued for driving more investment, demonstrating rural healthcare service quality and recruiting and retaining health workforce. However, strategies to build research capacity may need to focus on helping rural health services formulate research strategies, structures, governance and funding and to embed tools and planning processes in collaboration with research teams at rural universities. Such capacity could facilitate engagement and participation of diverse rural stakeholders in flexible ways. Addressing rural health research needs also requires robust place-based academic and practice networks across rural regions.

## 1 Introduction

Around the world, rural communities make up to 70% of the rural population and require health system strengthening for equity and universal health coverage (1). They also need tailored research evidence to drive learning organisations that can adopt rurally contextualized solutions (2). However, access to evidence to inform learning within the rural health system is impacted by an under-investment in rural health research. Competitive funding bodies in Australia, like other countries tend to use criteria and processes which tend to favor larger research teams, more controlled methods, larger datasets and specialized topics that are typically stronger in metropolitan research settings (3, 4). Furthermore, most research undertaken in rural areas is developed elsewhere with only a third involving local staff or local issues (5). Additionally, doctorally (PhD) trained researchers are under-represented in rural areas (15% relative to 29% population in Australia) (6) and rural clinicians and stakeholders have limited infrastructure and research time (7). As such, whilst rural health research may be important, crafting a feasible and effective strategy is complex and requires a relevant needs assessment.

Rural health research capacity building interventions have rapidly emerged in the last twenty years in Australia and overseas. However, few demonstrate that the interventions were designed based on a needs assessment with the end users (8–10). Some tend to draw from best practice educational and capacity building frameworks and theories to design interventions (8, 11, 12). Although research needs are likely to vary across rural health stakeholders, a ‘one size fits all’ strategy may not always work. Rural health research capacity building strategies may need to be centred on socially accountable responses tailored to what rural communities need.

Several research capacity building interventions focus on developing research capability (skills) of health sector employees via training modules and support for conducting research projects, some with additional funding, protected time and research coordination supports (9–11, 13–15). Most focus on rural clinicians, rather than the other key stakeholders such as rural health service executives and staff with roles contributing to rural healthcare access. Furthermore, some interventions centre on particular regions or single health organisations like hospitals or primary care services (9–11, 13, 14). Others cover specific health topics such as mental health and oncology (16, 17). There are few examples of interventions targeting research by rural early career medical doctors despite evidence suggesting that a lack of research opportunities in some fields like obstetrics and internal medicine could impact retention (18).

In addition, there is a paucity of literature on the impact of research capacity building interventions potentially because many interventions are still maturing. The available reported data about the impact of these interventions tends to describe participant (skills training and project enrolment and completion outcomes, presentations, publications and enrolments in research qualifications) and service changes without a control group (8, 13, 19–27). One study also found that providing research training for clinician-embedded researchers was useful for increasing research awareness but may not lead to behaviour change (26). The same study also suggested that enabling conditions like organizational systems developments and strategic partnerships were found to be other important elements for success (26). Other interventions involving clinician research support models noted that whilst participants achieved some publications and PhD enrolments, the research culture or clinical outcomes did not necessarily improve (19). Another formative evaluation of a multi-service cross-region research capacity building intervention further suggested that the barriers to rural health research participation amongst multi-hospital networks included mistrust and communication issues and the lack of a clear strategic plan about the changes expected (27). The aggregate findings hint at the complexity of rural health research capacity building and the need to more deeply understand needs to inform the development of a suitable program logic which can be effectively monitored for impact and outcomes over the implementation lifecycle.

Developing a needs-grounded, purposeful rural health research capacity building strategy is particularly important for rural health researchers who often work in small multidisciplinary teams and are pulled in many directions, covering a wide range of generalist topics (28). Crafting an evidence-informed strategy around rural health research capacity building might help rural health research teams to proactively plan and coordinate the use of resources across the region which covers hospital, primary care and wider rural stakeholder needs. The evidence around the intersecting needs, challenges and opportunities can also help to reflect on what theories are applicable within the strategy. With this background in mind, the aim of this research was to conduct a needs assessment of rural health research interests, barriers and enablers across rural hospitals and communities to inform the strategic directions for building rural health research capacity by distributed rural health research teams.

## 2 Materials and methods

### 2.1 Setting

The research was based in a rural university operating as part of a national network of universities funded by the Australian Government's Rural Health Multidisciplinary Training Program (RHMT) which covers all rural regions in Australia, to enable the training of rural medical students, provide placements and rural training opportunities for nursing and allied health students (in place since the mid-1990's). Our particular focus is in the Loddon Mallee and Gippsland region of Victoria, Australia. We also have a role in conducting rural health research and building research capacity of our local stakeholders. Many are clinicians and other rural stakeholders who might send emails, phone or request meetings for research support related to their work context or connecting with our rural university campus, or they may even want to enroll in research qualifications. Some may be involved in on-the-job and clinical teaching for our students around their substantive clinical role in the region or wanting to work out how to grow research or use data better as an organisation. However, despite out mandate to assist and our position as part of a national scale network, there is no systematic structure for units like ours to proactively respond to research capacity building, which leads to disorganised reactive approaches and challenges with how to use limited research resources. We did this research to understand the needs and inform the development of a clear strategy to address them.

### 2.2 Study design

This study was informed by a mixed-method research [MMR] design (29). MMR is the type of research where elements of qualitative and quantitative research approaches are combined to achieve a better understanding of the topic being studied (30). The strength of the mixed methods study lies in the understanding that the results generated from a combined method are greater than that those achieved from individual methods alone (31). The integration of quantitative and qualitative methods produces meta-inferences that may not otherwise be possible. A meta- inference, is ‘an overall conclusion, explanation, or understanding developed through an integration of the inferences obtained from the qualitative and quantitative strands’ (32). Since the needs and interests of rural health stakeholders were postulated to be diverse in both range and qualities, we believed MMR was suitable.

We employed a ‘convergent’ design where results from independently conducted quantitative and qualitative studies were compared and contrasted and we did this through regular research team meetings where we confirmed, disconfirmed, or explored how the quantitative and qualitative studies complemented each other (as data arose between October and November and then ongoing up until June 2026) (33). In this type of MMR design, once quantitative and qualitative data are collected and analysed separately, the results are compared and contrasted to examine if they converge or diverge. If the results diverge, an effort is made to explain the divergence (33).

Accordingly, we conducted a national survey along with interviews with rural health stakeholders within our rural and regional footprint to explore interests, challenges and opportunities for rural health research capacity building in more detail. We deliberately included all rural clinician types, stakeholders and both hospital and community settings in this study, to broaden the analysis of needs around rural health research.

#### 2.2.1 National survey

The survey was designed to gain insights into the range of skills, experience and priorities for clinicians and stakeholders to explore the spread of interests and level of activity. Our research team developed the survey to obtain information on research interest and things clinicians may prioritise by clinical area, career stage, sex, rurality and state based on the common requests for assistance that are sent to our research team. The survey was not based on pre-existing theoretical frameworks because there were not any that were specific enough to the rural context. It included 20 questions covering domains including research confidence, networking and engagement needs, project design and ideation, research execution and governance, publishing and dissemination, funding and resources, partnership and career development interests (Supplementary file 1). We piloted the online anonymous survey and then between October and November 2026, we circulated it via our national alliance of universities working under the RHMT, the Federation of Rural Australian Medical Educators (FRAME) (34) and Australian Rural Health Educators Network (ARHEN) covering rural nursing and allied health training (35). We also sent it out to all stakeholders known to the research team asking that they snowball to ensure we reached a wider sample. All participants received written information about the study and the survey was completely anonymous and completed through a link in the email which led to Google Forms. At the end of the survey, participants could nominate for a research interview if they were based in the Loddon Mallee or Gippsland regions and if so, informed written consent and basic eligibility information and contact details were collected. There was no funding to participate. Data from the survey were analysed descriptively.

#### 2.2.2 Interviews

Those who nominated for the interview between October and November 2026, were contacted by the research team via email to arrange an interview online, audio-recoded via Zoom or face-to-face, and recorded using a handheld device. A maximum of three participants could attend each interview as some participants had more than one informant, they wanted to include due to topic interest and perceived relevance. Up to one-hour semi-structured interviews aimed to build on the survey and ask four simple questions, exploring the respondent's context, role, position, region and career stage and previous research experience, why they want to engage with rural health research and why? What help they need from rurally based researchers, and what help is needed from rural services and organisations. The questions were broad to allow open dialogue without pre-conceived ideas around perspectives, challenges or solutions. The interviewer (BOS) was consistent throughout to support consistency and the interviewer discussed emerging findings with the research team to formulate areas for prompting in future interviews. Some of the prompts involved getting more information about their motivations and more specific information about the help they needed from different parties and why. The audio-recordings were de-identified and assigned a unique ID. We used E for executive representing someone from hospital or community and from clinical or non-clinical roles, C for clinician, whether primary care or specialist of any discipline. We also labelled sex and career stage, to aid our interpretation of the data. De-identified audio recordings were transcribed using Turboscribe software.

Thematic analysis was applied to the transcripts (36, 37). Thematic analysis can be used for inductive and deductive analyses to capture both explicit and underlying meaning. Initially, all researchers read the transcripts and discussed high-level themes (36). Coding was initially deductively informed based on an emerging hypothesis that there might be differences between community, hospital, clinician and executive perspectives of the system. This was not founded in the literature of critical frameworks, however it was postulated based on the experience of the research team of rural settings where resources and interests may vary depending on employment models and leadership levels. However, after the interviewer (BOS) and other researchers (LW and AI) independently undertook the initial coding, it was discussed as a research team that there was minimal differences between the former two, so community and hospital coding were collapsed into two (clinician and executive differences) where there were perceived differences in the results. The research team met regularly and used double-coding and discussion around how to label themes and sub-themes to represent the meaning of the data. This involved gradually collapsing codes into the most common patterns in the data, whilst noting points of variation. Other deductive themes followed the research questions (interests, challenges, opportunities), allowing inductive codes to emerge through reading and re-reading and discussing as a research team, reflecting on other research and confirming the meaning of the codes.

#### 2.2.3 Reflexivity

Our research team was situated within a funded program specifically required to engage in regional research capacity building so we were embedded in the phenomenon under study. Managing a broad portfolio of roughly 150 projects across a geographically dispersed, multidisciplinary network forces our team of around 7–8 FTE PhD-trained researchers meant we conducted this research and analysis through a pragmatic lens to inform rural health research units and in doing so, we may have missed theoretical interpretations of the data (38). To manage suitable coding, theme development and interpretation of the findings given our dual role, we remained consciously reflexive about how our mandate to build research capacity might subtly favour actionable, applied solutions during our data analysis, and we actively use team-based debriefing sessions and engaged an independent student researcher and a multidisciplinary research team, to mitigate potential confirmation bias.

This study had ethical approval from Monash University HREC, Project ID: 49681, 13/10/2025.

## 3 Results

### 3.1 National survey

Forty-six responders commenced and 40 completed the survey. The majority of commencing respondents (50%) were from Victoria, 20% New South Wales and 15% Queensland and the remainder from other states and territories (Table 1). Most (39%) were also in the 45–54-year age group, all worked in non-metropolitan areas with 37% from a smaller rural town, had studied medicine (67%), had trained as a clinician locally (59%), with over 20 years’ experience (44%) and worked in a hospital (48%). We were not able to assess response bias as we did not know the denominator, however the data may have missed non-medical clinicians and certain regions and should be thus be treated indicatively. See Table 1.

**Table 1:** Demographic characteristics of survey respondents (*N* = 46).

Characteristic	Category	Number (%)	Characteristic	Category	Number (%)
Age group			Field of Education^a^	Medicine	31 (67)
	25–34	2 (4)		Nursing	3 (7)
	35–44	11 (24)		Allied Health	4 (9)
	45–54	18 (39)		Health Administration	6 (13)
	55–64	10 (22)		Public Health	6 (13)
	65+	5 (11)		Other	6 (13)
Gender	Male	20 (44)	Overseas trained	Yes	13 (28)
	Female	25 (54)		No	27 (59)
	Prefer not to say	1 (2)		Not applicable	6 (13)
Indigenous status	No	46 (100)	Clinical experience	In training	3 (6)
State of residence	VIC	23 (50)		<10 years	7 (15)
	NSW	9 (20)		10–20 years	9 (20)
	SA	3 (7)		>20 years	20 (44)
	WA	2 (4)		Not applicable	7 (15)
	QLD	7 (15)	Current employment^a^	Hospital	22 (48)
	TAS	2 (4)		Aboriginal health organisation	4 (9)
Work location	MMM2	16 (35)		Private practice	17 (37)
	MMM3	13 (28)		Community health agency	3 (6)
	MMM4–7	17 (37)		University	20 (44)
				Other	7 (15)

MMM, Modified Monash Model; MMM2, Large regional centre>50,000 population; MMM3, smaller regional town = 15–50,000 population; MMM4–7, rural town < 15,000 (Australian Government Department of Health Disability and Aging, 2026).

a Some respondents had completed more than one field of education and were employed in more than one organisation.

Of 40 who completed the survey, ninety percent reported an interest in rural health research in some form with 50% reporting no formal research qualifications. Over 50% reported being research active, although only a quarter of respondents reported that their role required them to undertake research. Sixty five percent of respondents reported having been involved in research previously and 70% expressed an interest in being actively involved in research as part of their ongoing career. Ninety five percent of respondents believed that rural communities could benefit from research focused on rural health issues.

Of the 40 respondents, 95% rated getting to know researchers in their region and the projects they were working on as important or very important. 85% rated joining research projects in the region where they could play a role as important or very important. However, proportion of those who rated needing help with tasks like shaping and leading their own research steadily decreased from 62%. See Figure 1.

**Figure 1:**
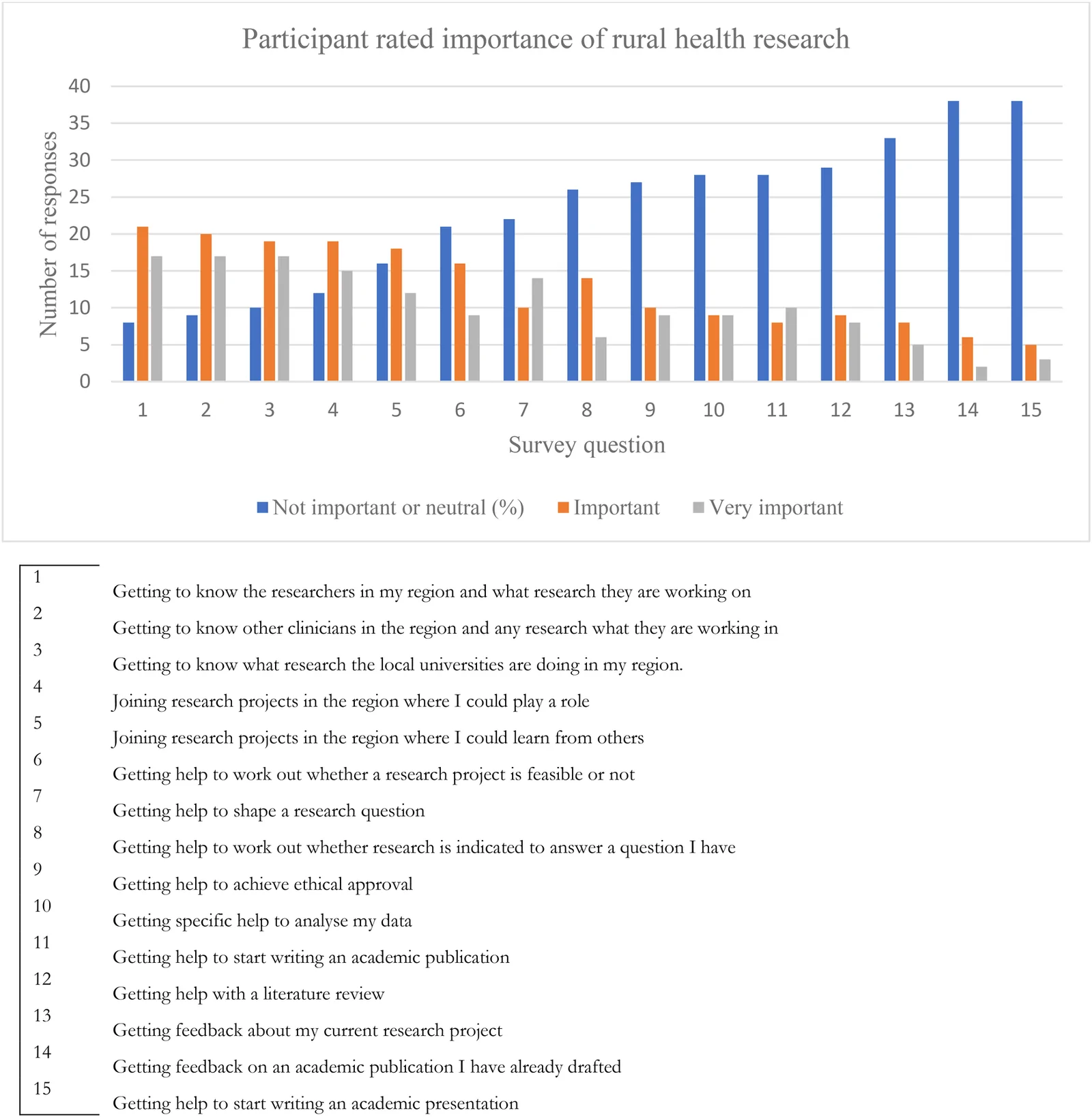
Stakeholder needs and interest in rural health research (*N* = 40).* six respondents did not complete this section of the survey.

### 3.2 Interviews

A total of 26 individuals participated across fifteen one-to-one interviews, four one-to-two interviews and one one-to-three interview. Participants included eighteen executives and eight clinicians, 20 of whom were from the Loddon Mallee region and six from the Gippsland region. Further details of participants are given in Table 2.

**Table 2:** Demographic characteristics of interview participants (*n* = 26).

*Characteristic*	*Number (%)*	*Characteristic*	*Number (%)*
Sex		Practice location	
Male	12 (46)	Hospital clinician	4 (15)
Female	14 (54)	Community clinician	2 (8)
**Region of practice**		Hospital & community clinician	2 (8)
Loddon Mallee	20 (77)	Hospital executive	7 (27)
Gippsland	6 (23)	Community executive	11 (42)
Primary role		Career stage ^a^	
Clinicians (total)	8 (31)	Mid-career stage	25 (96)
Executives (total)	18 (69)	Late-career stage	1 (4)

a Career stage: Mid-career > 10 years to retire; Late-career < 10 years to retirement; There were no respondents in the early stage of their career (<10 years since their initial qualification).

The main themes that emerged from the data were: 1. Value of rural health research, 2. Barriers to rural health research and 3. Enablers of rural health research. Each of them varied slightly between rural executives and clinicians which we indicate in our description of the results but they were very consistent across hospital and the wider community. The themes and subthemes are presented in Table 3.

**Table 3:** Themes generated from the interviews and their relevance to stakeholders .

Themes	Relevant to Executives	Relevant to Clinicians
Value of rural health research		
Drive system investment	✓	
Informs quality services	✓	
Informs quality care		✓
Recruit and retain workforce: rural work attractive	✓	✓
Barriers to rural health research		
Priorities and funding of funder/executive	✓	✓
Organising it structurally – resources and processes unclear and need tools^a^	✓	✓
Respecting limited time and resources, clinical service demand		✓
Enablers of rural health research		
Organised around community need	✓	
Research planning and pathways and tools *		✓
Research teams, leadership and academic networks	✓	✓

a Clinicians noted gaps in access to tools included library, ethics committees and data as “resources, processes and need tools” under this sub-theme.

#### 3.2.1 Value of rural health research

Executives were most interested in system investment which could be important due to the resource limitations in rural health organisations: “get grant funding coming in” (2EGMFM) with research also valued for building: “evidence that will actually drive investment in rural health systems” (8ELMFM).

From a quality perspective executives noted the value of research for informing organizational quality: “we look at things that are really important to our workforce and to our organisation” (2EGMFM). They wanted: “really practical research that sort of spells out the value of what you're doing…brings design to the table to the good models” (7ELFFM). For clinicians, quality was valued as a chance to show service quality in rural contexts rather than only having metropolitan evidence: “to have guidelines that work for us, we need an evidence base that comes from us as rural clinicians and our rural patients” (10CLFM).

Both executives and clinicians were strongly in favor of the impact of research and recruitment and retention, for organizational benefits and making rural healthcare attractive and rewarding as a career option. Executives viewed research as helping to attract high caliber staff growing careers locally: “creating a brand where we can recruit highly capable people from high pedigree coming to this institution” (8ELMFM). Part of this involved engaging junior doctors: “We get our medical students involved in our research [it] helps us look more attractive as an organisation when they're thinking about their career path” (2EGMFM). Clinicians viewed recruitment and retention as providing careers on par with metropolitan areas, opportunities to stay in one place and doing meaningful work: “research … one of those things that can be offered and can be developed as reasons for people to stay around and work on meaningful projects” (12CLMM).

#### 3.2.2 Barriers

Over and above interests in rural health research, or opportunities to do it, was the theme of challenges which was the most commonly discussed. There were three main sub-themes summarizing the challenges including: 1) Priorities and funding of funder/executive; 2) Organizing it structurally; 3) Respecting limited time and resources. The executive and clinicians both relayed perspectives on the three main sub-themes, suggesting they matter to both groups. The strongest reported challenge for executives and clinicians was the operationalization of research through the rural health system with executives noting challenges with connecting strategy and operational activity within the resourcing available: “… the research and innovation team have been shuffled around a lot …” (20_ELFL_hospital) and “we can have it in abstract plans. But it's hard to operationalize…rural have different resourcing” (1EGFM_hospital). Clinicians identified not having clear processes for engaging with research, within healthcare structures: “…the issue is if then people come with bright ideas, I have nowhere to send them… I've got junior doctors …but I have nowhere to put them” (10CLFM_community-hospital). Another commented that data infrastructure is under-developed for research: “I had the idea…[but] how could we measure every single patient that comes in, because our EMR [electronic medical record] is crap…we had all these paper-based forms” (3CGFM_hospital). Other perceived issues were clinician and executive access to a library: “I can't get through the gate to see, ‘Oh somebody's already done that study! great’, you know, Wonderful, moving on, next idea” (10CLFM_community-hospital). Both also commented on ethics committee access with executives noting the challenge of getting one: “One of the key gaps is we're not formally attached to an ethics committee” (8ELMFM_hospital) and a clinician noting the efficiency of the process being poor: “you get pinged back from research, from the ethics application too many times, why would you bother?” (3CGFM_hospital).

The next strongest perceived challenge was the lack of respect for limited time and resources which was more heavily weighted by rural clinicians, than executives. Comments by executives suggested that clinicians may have limited free time compared with their counterparts in the city: “their non-clinical time is like five hours. The guys in [city], non-clinical time is 40” (1EGFM_hospital). There were also perceived challenges in finding spare time: “We've all got very limited resources there are a few assumptions being made about how easy it is to do research” (20_ELFL_hospital). For clinicians: “I haven't done a publication. I haven't done anything because all I'm doing is seeing patient after patient … whatever project I want to do, I don't have the resources to go into it.” (11CLMM_hospital). Research was expected around high clinical demands and perceptions of smaller clinical teams: “in a department that requires five consultants, we've got two. So, you just don't have the time…” (14CLMM_hospital). When committing to research, clinicians also perceived a need to be wary that it didn't interrupt their main role: “if it starts taking up a lot of my FTE, I can't participate because that's not why I get paid here” (10CLFM_community-hospital). To manage costs, clinicians tended to focus on clinical trials because they had funding: “investigator- initiated research is usually unfunded” (11CLMM_hospital).

#### 3.2.3 Enablers

There were three major enablers as sub-themes: 1) Organizing research around community need; 2) Research planning, pathways and tools and; 3) Research teams, leadership and academic networks. For organizing research around community need, executives perceived that regional priorities could be shared: “they're admiring the problem, but they've all got the same problem. But not one of them has said, hey, do you want to hang out?” (1EGFM_hospital). They went on: “research is good when it's not just in one service, but it's on an issue that affects everyone” (1EGFM_hospital). Firstly, strategic planning for research and research pathways was considered essential: “So, as an organisation, we have built research into our strategic plan” (9ELMMM_community) as was operational planning: “we have to come up with a very clear plan, business case… people who want to, who are passionate about research in whatever the area is, are supported to do it” (1EGFM_hospital). An ethics committee and data infrastructure were other developments clinicians said would be enabling: “I would love to have an ethics committee”…to be at the end of we do need one. (10CLFM_community-hospital).

The strongest perceived opportunity for executives and clinicians was around research teams, leadership and networks. Executive noted the importance of spotting clinical champions: “that first group of people are going to be the champions…they're going to be looking for their fans and supporters to come and join them” (1EGFM_hospital). The perception was that research literacy and teams and programs were important: “we don't need a building that's dedicated to research. We need the people that all understand what it means …to grow a research program.” (20_ELFL_hospital). Clinicians noted the value of students for buffering their time: “students because they have come in and been able to kind of just do a project like, it's just the thing that someone does” (12CLMM_hospital-community). They saw the value of formalizing connections with academic units and providing co-appointed roles: “all of my GP supervisors, when I was training, were actually cross-appointed academics” (10CLFM_community-hospital).

### 3.3 Consolidated findings

The findings from the national survey and interviews aligned in showing a strong interest in the value of rural health research. However, whilst suggesting that rural stakeholders wanted to be aware of research that was being done locally, the results suggest that they preferred to be involved in a supportive role rather than in a leading role of undertaking research themselves. The reason for this finding is perhaps the range of systems-level barriers to research that were highlighted in the qualitative findings. These could be things like the structural organisation and the amount of time rural clinicians have to give to patient care within smaller rural clinical teams and the lack of capacity of executives to employ more staff to relieve them. Specifically, the finding that systems, processes and networks around opportunities to be part of team-based research suggests there is a possibility that rural health stakeholders could increasingly engage in research and build their skills, if the right supports were in place.

## 4 Discussion

Our research provides a grounded needs assessment of rural health research capacity building which encompasses clinicians in hospitals and the community and executives in health services and other rural stakeholder organisations. The findings suggest that there is strong perceived value of engaging in rural health research. The small survey indicated that around half of rural respondents had some research experience and wanted research to be part of their role but lacked confidence or didn't intend on pursuing it on their own. Rather it was common to want more information on the type of research being done, and on how to get involved in research projects. This suggests that systems development, tools and networked connections with academics in the region was needed so that rural executives and clinicians can access research and work with researchers at a level of their choosing.

When comparing responses across the stakeholder groups interviewed, there were nuanced interests depending on their position in the system. Importantly, clinicians may be drawn to research which helps their patients to get high quality care in rural contexts. This motivator is important to consider when thinking about engaging clinicians in research which could motivate them and sustain interest. This suggests that relevant datasets for these clinicians might need to include measures of rurality, consider rural models, patient demographics and epidemiological profiles as well as being easy to access. It is possible that metropolitan research centres could assist in building some of this infrastructure into their datasets including asking rural clinicians what questions they have and working through how the data can answer rural community needs for the questions that interest them.

The interviews targeted fairly open-ended questions but it was intriguing as to how little attention respondents gave to learning research skills compared with their demand for more responsive research systems to enable them to take part in research processes. The latter was almost the only issue discussed and it may relate to a perception amongst respondents of having limited agency over research participation when systems factors made it impossible or unsustainable. The finding suggests that relevant theories for rural health research capacity building need to cover multiple systems developments and not just educational approaches. Further, they might be more successful if they encouraged communities of practice, by learning on the job rather than doing individual projects. This was well articulated by Schmidt et al. (15, 39) who have suggested that while providing protected time, funding and mentorship could build individual clinical research capability, structural factors also need to be addressed in order to build rural health research capacity that generates meaningful improvements in rural health.

A major barrier mentioned by clinicians and also discussed by executives was the time to do research, with both parties noting that research is time consuming and non-feasible as part of their roles. This suggests that rural health research capacity building approaches need to give particular attention to ensuring that there are enough PhD trained rural health research staff in rural teams to enable not just supporting rural clinicians, but also doing a large part of their research and promoting learning on the job. This is challenging for small rural research teams with high workloads (38). To address this, it might be necessary to invest more in PhD-trained health researchers in rural regions who play roles related to engaging rural health stakeholders and networks in research opportunities. Larger, clean and routinely collected data might also help to reduce the time intensive labour of rural health researchers negotiating individual topics, methods and ethics, with rural clinicians. However, caution would be needed before targeting single datasets or streamlined methods as wider research suggests that rural health research needs to be socially accountable to emerging needs and interests in the system, rather than fixed in nature (38).

The interviews identified that much of the rural health research infrastructure may be quite under-developed, with limited routine data, library access, inefficient and inaccessible ethical review processes, and few project planning supports embedded. A major solution recognised by both executives and clinicians could be building a research strategy aligned with funding, investing in operational processes which can be embedded over time and promoting leadership, networks and partnerships (5). This suggests that each rural health organisation may need to link up with rurally-based universities in a region-wide approach where research priorities are shared and co-resourced by multiple agencies. The national networks of ARHEN and FRAME provide a unique way to scale this approach up nationally. Phased plans may be needed to build the necessary operating infrastructure and processes to get involved, in partnership with rural health research units. This is a longer-term approach which might align with the results of the survey, by helping the entities involved to share information and invite collaboration in research planned or underway. However, to mitigate overburdening existing research staff, prioritisation of the research is likely to be essential, such as checking that research ideas are relevant, feasible and effective.

Our study had limitations. The study sample was small and only provided indicative data, with no response rates as snowballing was used and the denominator was not known. The interviews were also confined to two rural regions and up to 26 interviewees because this research was unfunded, however we had a sense that we had reached consistency in the emerging themes, with no new themes arising at the time we concluded the interviews. Interviewees self-selected and focused on clinicians and executives that the researching university is often asked to support, however, the findings could have been enhanced by including consumer and First Nations perspectives. Including the latter groups may have been possible with more time and funding. The views are limited to the regions studied but views may differ in other areas. Participants may have emphasized issues that they hoped the university could address and moderated criticism of existing research capacity because of perceived professional relationships or power differentials between the university and their organization. They could have also focused on topics they believed would be of particular interest to university researchers. The level of engagement was strong with many participants enthusiastically contributing insights about the topic, but the data may not represent the average view including people without motivations to do rural health research, so the evidence should be interpreted with caution.

## 5 Conclusion

This study provides novel insight into the values, challenges and opportunities for clinicians and executives to embed rural health research within their organisations. While the value of research for rural communities was almost unanimously supported, and executives and clinicians may be interested, there is a wide range of rural health research strategies, governance and structures, processes and research networks that may need development to meet the needs of rural regions. Our study provides a useful foundation for future discussions regarding the design of rural health research capacity building approaches, particularly those which address the multilevel needs of different stakeholders across a region rather than individualized approaches focused on skills training in one group or institutional setting. In doing so, the study can help inform the development of a more robust and sustainable rural health research activity, in an effort to inform rural health systems and build learning organisations.

## Data Availability

The datasets presented in this article are not readily available because the ethical approval under which the data were collected does not permit sharing of the raw data. Requests to access the datasets should be directed to Belinda O'Sullivan E: belinda.osullivan@monash.edu.
