# Supplementary material for: Needs assessment to shape rural health research capacity building strategies: a mixed methods study
Source: Front Med (Lausanne). 2026 Oct 28;13:1932638. doi: 10.3389/fmed.2026.1932638 (PMC13751406; doi:10.3389/fmed.2026.1932638)
Supplement: Supplementary file 1 [file Table1.docx]

Supplementary File 1: Survey Questionnaire

** Indicates required question*

**Demographics and Current Role**

**1. What state do you live in? ***

*(Select one option)*

[ ] VIC

[ ] NSW

[ ] SA

[ ] WA

[ ] NT

[ ] QLD

[ ] TAS

[ ] ACT

[ ] Other

**2. Where is your main location of work? (MM = Modified Monash Model)**

*(Select one option)*

[ ] MM 1 (metropolitan)

[ ] MM 2 (large regional centre >50,000 population)

[ ] MM 3 (smaller regional town 15,000–50,000 population)

[ ] MM 4–7 (rural town <15,000 population)

**3. Where do you currently work? (Tick all that apply) ***

*(Select all that apply)*

[ ] Hospital

[ ] Aboriginal Community Controlled Health Organisation

[ ] Private practice

[ ] Community health agency

[ ] University

[ ] Stakeholder agency

[ ] Other

**Research Interest & Activity**

**4. Do you think rural communities could benefit from research focused on rural health issues?**

*(Select one option)*

[ ] Yes

[ ] No

[ ] Maybe

**5. Do you have an interest in rural health research? ***

*(Select one option)*

[ ] Yes, as a leading researcher

[ ] Yes, as a supporting researcher

[ ] Yes, as a user of rural health research

[ ] No

[ ] Not sure

**6. Are you research active currently? (defined as having an active research project planned or underway) ***

*(Select one option)*

[ ] Yes

[ ] No

**7. Do you have any formal qualifications in research? ***

*(Select one option)*

[ ] Honours

[ ] Masters

[ ] PhD/Doctorate

[ ] No

**8. Do you want to be actively researching, as part of your ongoing career? ***

*(Select one option)*

[ ] Yes

[ ] No

[ ] Not sure

**9. Does your role require you to do research?**

*(Select one option)*

[ ] Yes

[ ] No

[ ] Maybe

**Clinical Training and Experience**

**10. Did you complete your basic clinical training overseas? ***

*(Select one option)*

[ ] Yes

[ ] No

[ ] Not a clinician

**11. Are you training/trained in: medicine, nursing, allied health or health administration? (Tick all that apply)**

*(Select all that apply)*

[ ] Medicine

[ ] Nursing

[ ] Allied health

[ ] Health administration

[ ] Public health

[ ] None of the above

**12. When did you complete your clinical training? ***

*(Select one option)*

[ ] Still training

[ ] In the last 10 years

[ ] In the last 10–20 years

[ ] Over 20 years ago

[ ] Not applicable

**13. Are you currently clinically active? (working at least some FTE clinically) ***

*(Select one option)*

[ ] Yes

[ ] No

[ ] Not applicable

**14. Have you previously done any research projects where you were a co-investigator or leader? ***

*(Select one option)*

[ ] Yes

[ ] No

[ ] Not sure

**Research Confidence and Skills**

**15. To what extent do you feel confident to commence research on a rural health topic? ***

*(Select one option on a scale from 1 to 5)*

| **1 - Not confident** | **2** | **3** | **4** | **5 - Very much confident** |
| --- | --- | --- | --- | --- |
| [ ] | [ ] | [ ] | [ ] | [ ] |

**16. To what extent do you think you have the skills to do research on rural health topics? ***

*(Select one option on a scale from 1 to 5)*

| **1 - Not confident** | **2** | **3** | **4** | **5 - Very much confident** |
| --- | --- | --- | --- | --- |
| [ ] | [ ] | [ ] | [ ] | [ ] |

**Research Needs and Priorities**

**17. What are your needs and interests in rural health research? ***

*(Please rate each item on a scale from 1 to 5)*

*Likert Scale: 1 = Not at all important | 2 = Not important | 3 = Neutral | 4 = Important | 5 = Very important*

| **Research Activity / Need** | **1 (Not at all important)** | **2 (Not important)** | **3 (Neutral)** | **4 (Important)** | **5 (Very important)** |
| --- | --- | --- | --- | --- | --- |
| **Networking & Engagement** |  |  |  |  |  |
| Getting to know what research the local universities are doing in my region | [ ] | [ ] | [ ] | [ ] | [ ] |
| Getting to know the researchers in my region and what research they are working on | [ ] | [ ] | [ ] | [ ] | [ ] |
| Getting to know other clinicians in the region and any research they are working on | [ ] | [ ] | [ ] | [ ] | [ ] |
| Joining research projects in the region where I could play a role | [ ] | [ ] | [ ] | [ ] | [ ] |
| Joining research projects in the region where I could learn from others | [ ] | [ ] | [ ] | [ ] | [ ] |
| **Project Design & Ideation** |  |  |  |  |  |
| Getting help to shape a research question | [ ] | [ ] | [ ] | [ ] | [ ] |
| Getting help to develop a multi-year research program (integrated program of work on a focus area) | [ ] | [ ] | [ ] | [ ] | [ ] |
| Getting help with a literature review | [ ] | [ ] | [ ] | [ ] | [ ] |
| Getting help to work out whether research is indicated to answer a question I have | [ ] | [ ] | [ ] | [ ] | [ ] |
| Getting help to work out whether a research project is feasible or not | [ ] | [ ] | [ ] | [ ] | [ ] |
| Getting research done to tick a milestone requirement | [ ] | [ ] | [ ] | [ ] | [ ] |
| **Execution & Governance** |  |  |  |  |  |
| Getting feedback on a research project I'm doing | [ ] | [ ] | [ ] | [ ] | [ ] |
| Getting help to achieve ethical approval | [ ] | [ ] | [ ] | [ ] | [ ] |
| Getting help to qualify as an academic supervisor (i.e., supervise Master's/PhD students) | [ ] | [ ] | [ ] | [ ] | [ ] |
| Getting feedback about my current project progress | [ ] | [ ] | [ ] | [ ] | [ ] |
| Getting specific help to analyse data | [ ] | [ ] | [ ] | [ ] | [ ] |
| **Publishing & Dissemination** |  |  |  |  |  |
| Getting help with writing an academic publication | [ ] | [ ] | [ ] | [ ] | [ ] |
| Getting feedback on an academic publication I have already drafted/started | [ ] | [ ] | [ ] | [ ] | [ ] |
| Getting feedback on an academic presentation | [ ] | [ ] | [ ] | [ ] | [ ] |
| **Funding & Resourcing** |  |  |  |  |  |
| Finding out where I can get grant funding | [ ] | [ ] | [ ] | [ ] | [ ] |
| Getting research assistants or staff to help with my project | [ ] | [ ] | [ ] | [ ] | [ ] |
| Getting help with writing a grant application | [ ] | [ ] | [ ] | [ ] | [ ] |
| Getting feedback on a grant application I have already drafted | [ ] | [ ] | [ ] | [ ] | [ ] |
| **Partnerships & Career Development** |  |  |  |  |  |
| Finding a university partner to collaborate on a rural project | [ ] | [ ] | [ ] | [ ] | [ ] |
| Joining a research team / local clinical group doing rapid reviews to inform rural health topics | [ ] | [ ] | [ ] | [ ] | [ ] |
| Finding paid adjunct academic advisor positions in a rural region | [ ] | [ ] | [ ] | [ ] | [ ] |
| Finding paid research positions in a university or health service | [ ] | [ ] | [ ] | [ ] | [ ] |
| Learning research skills (micro-credential, don't want a full qualification) | [ ] | [ ] | [ ] | [ ] | [ ] |
| Doing research to get a PhD or other academic qualification in a rural area | [ ] | [ ] | [ ] | [ ] | [ ] |

**Personal Demographic Profile**

**18. What is your gender? ***

*(Select one option)*

[ ] Male

[ ] Female

[ ] Prefer not to say

[ ] Other: ____________________

**19. Do you identify as a First Nations person? ***

*(Select one option)*

[ ] Yes

[ ] No

**20. What is your age group (years)? ***

*(Select one option)*

[ ] 18–24

[ ] 25–34

[ ] 35–44

[ ] 45–54

[ ] 55–64

[ ] 65+

*End of Survey*
